# Intradural Extramedullary Thoracic Spinal Paraganglioma in a Young Adult: A Case Report

**DOI:** 10.7759/cureus.102734

**Published:** 2026-01-31

**Authors:** Serge E Bambule, Tarik Chekrine, Mouna Bourhafour, Medhi Karkouri, Souha Sahraoui

**Affiliations:** 1 Medical Oncology, Mohammed VI Center for Cancer Treatment, University Hospital Center Ibn Rochd, Faculty of Medicine and Pharmacy, University Hassan II, Casablanca, MAR; 2 Internal Medicine, Kinshasa University Clinics, Faculty of Medicine, University of Kinshasa, Kinshasa, COD; 3 Radiotherapy, Mohammed VI Center for Cancer Treatment, University Hospital Center Ibn Rochd, Faculty of Medicine and Pharmacy, University Hassan II, Casablanca, MAR; 4 Pathology, University Hospital Center Ibn Rochd, Faculty of Medicine and Pharmacy, University Hassan II, Casablanca, MAR

**Keywords:** case report, intradural extramedullary tumor, paraganglioma, radiotherapy, spinal cord compression, thoracic spine

## Abstract

Spinal paragangliomas are rare, typically benign neuroendocrine tumors, exceptionally uncommon in the intradural extramedullary thoracic region. Their diagnosis is particularly unusual in young adults. We report the case of a 27-year-old patient who presented with progressive paraparesis due to spinal cord compression caused by a 36 × 17 mm mass at the D3-D4 level, identified on MRI. Surgical resection was performed. Pathological examination confirmed the diagnosis of spinal paraganglioma, supported by immunohistochemistry (positive for chromogranin A, synaptophysin, and focal expression of S-100 protein). Given the risk of local recurrence, adjuvant radiotherapy was administered. Clinical outcome was favorable, with complete recovery of motor deficits following functional rehabilitation. Radiological follow-up at one year post-radiotherapy showed no signs of recurrence.

## Introduction

Paragangliomas are rare neuroendocrine tumors with an estimated global incidence of 2 to 8 cases per million [1]. No specific data on African continental incidence are available; African data are extrapolated from global figures. These tumors originate from paraganglion cells derived from the neural crest and develop along vasculo-nervous axes [1]. Approximately 70% are benign, with malignancy defined by the presence of metastases [2]. At the spinal level, the classic form is intradural extramedullary, predominantly affecting the filum terminale and cauda equina [1,3]. Thoracic or cervical locations are far rarer and may radiologically mimic meningiomas or schwannomas, making preoperative diagnosis challenging [4,5]. Confirmation relies on histopathological examination, often supplemented by immunohistochemistry. Treatment is based on complete surgical resection, the primary objective, and may be complemented by adjuvant radiotherapy to reduce the risk of recurrence [6]. 

We report a rare case of thoracic spinal paraganglioma in a young adult, highlighting the rarity of this location, diagnostic challenges, and the importance of multidisciplinary management combining surgery and radiotherapy.

## Case presentation

Patient information

A 27-year-old man with no significant medical history presented with progressive lower limb weakness, evolving over three months into paraparesis. There were no sensory disturbances, sphincter dysfunction, or systemic symptoms. General condition was preserved.

Clinical findings

Neurological examination revealed bilateral lower-limb weakness graded 3/5 on the Medical Research Council scale. Sensory examination, deep tendon reflexes, and sphincter function were normal.

Timeline

In December 2023, spinal MRI identified an intradural extramedullary mass at D3-D4. Surgical resection was performed. In June 2024, the postoperative MRI showed persistent poorly defined subarachnoid infiltration from D3 to D8 with hyper signal, suggesting residual disease. 18-fluorodeoxyglucose (FDG) PET demonstrated no hypermetabolic lesions. In July-August 2024, adjuvant radiotherapy was delivered, and in August 2025, follow-up MRI confirmed absence of recurrence.

Diagnostic assessment

**Figure 1:**
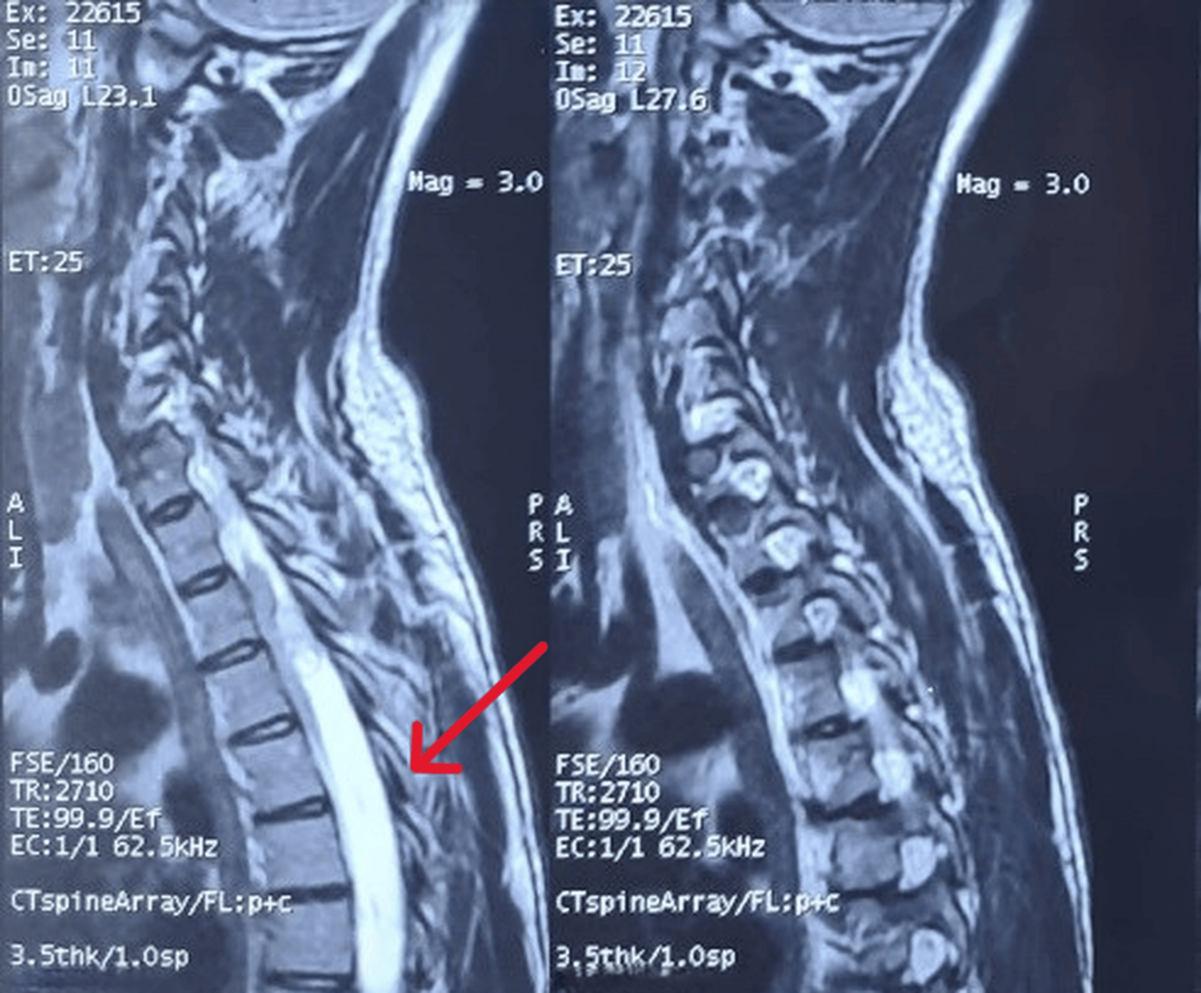
Preoperative thoracic spine MRI (T2-weighted fast spin-echo (FSE) sequence) showing a 36 × 17 mm intradural extramedullary mass at the D3–D4 level, associated with a focal intramedullary T2 hyperintensity indicating spinal cord involvement.

**Figure 2:**
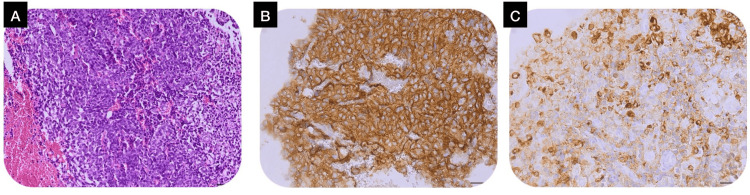
Histopathological samples from paraganglioma: A) Hemorrhagic tumor proliferation arranged in layers and focally in clusters, hematoxylin-eosin stain, ×20, scale bar = 50 microns. B) Positive expression of synaptophysin, hematoxylin-eosin stain, ×40, scale bar = 50 microns. C) Positive expression of chromogranin A, hematoxylin-eosin stain, ×40, scale bar = 50 microns.

MRI demonstrated a well-defined intradural extramedullary lesion at the D3-D4 level measuring 36 × 17 mm, causing significant spinal cord compression (Figure 1). Histopathological examination revealed a hemorrhagic tumor proliferation arranged in layers and focally in clusters. Immunohistochemistry showed diffuse positivity for chromogranin A and synaptophysin, with focal S-100 protein expression, confirming the diagnosis of paraganglioma despite the atypical histological appearance (Figure 2). Plasma free metanephrines and normetanephrines were within normal limits, consistent with a nonfunctional tumor.

Diagnosis

Diagnosis was a nonfunctional intradural extramedullary thoracic spinal paraganglioma.

Therapeutic interventions

**Figure 3:**
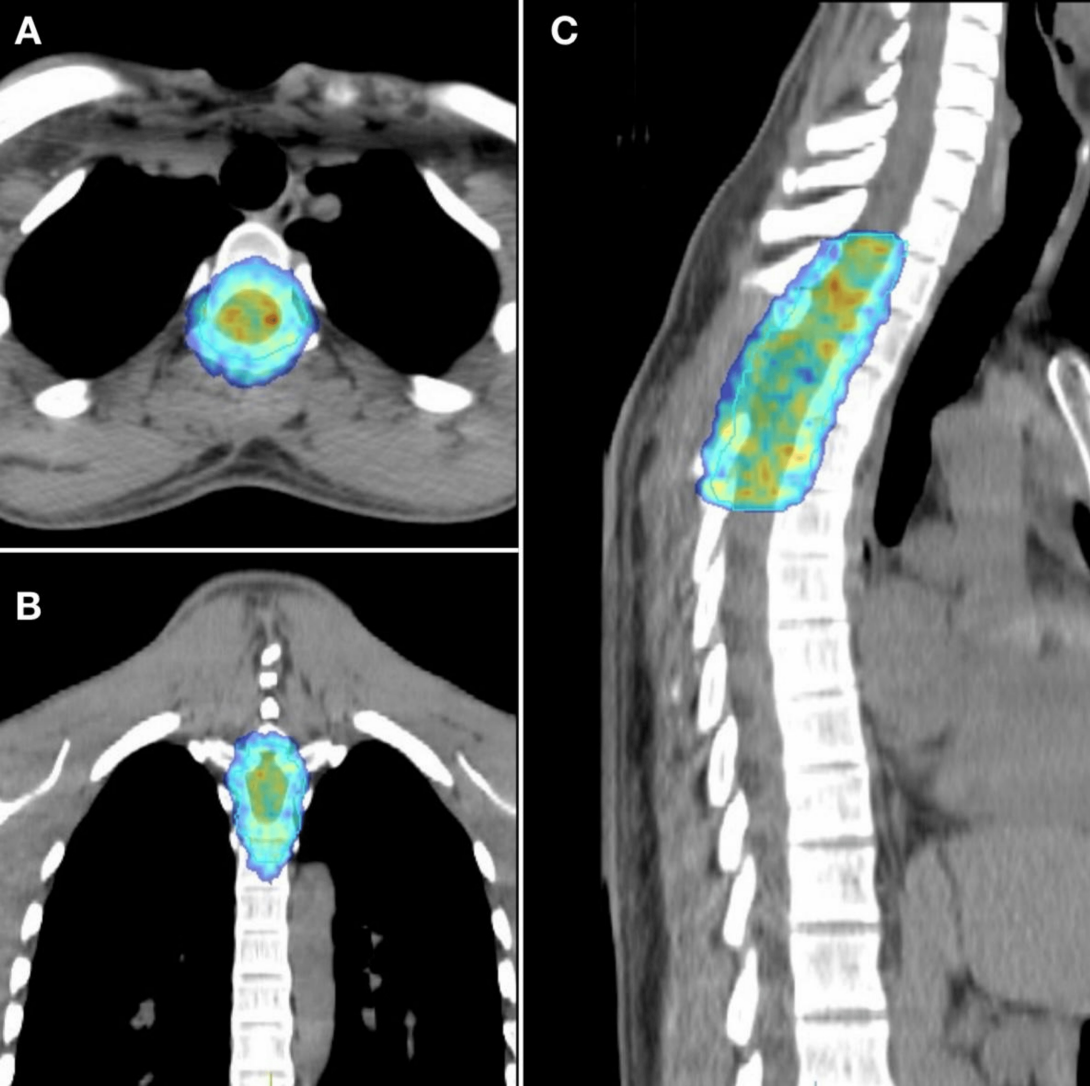
Volumetric modulated arc therapy (VMAT) radiotherapy to the D1–D4 region, shown on (A) axial, (B) coronal, and (C) sagittal views.

Surgical excision of the tumor was achieved, followed by functional rehabilitation. Given the persistence of poorly defined subarachnoid infiltration with hypersignal suggesting residual disease on postoperative MRI, adjuvant external beam radiotherapy was administered using volumetric modulated arc therapy (VMAT), targeting the D1-D4 region with a total dose of 45 Gy delivered in 1.8 Gy fractions (Figure 3).

Follow-up and outcomes

The patient experienced complete recovery of motor function. At 12-month follow-up post-radiotherapy, clinical examination was normal and spinal MRI showed no evidence of local recurrence.

Patient perspective

The patient reported satisfaction with the outcome and relief after regaining full mobility, acknowledging the importance of continued follow-up. Informed consent was obtained.

## Discussion

Intradural extramedullary spinal paragangliomas, though rare, can occur at any age. The typical age of onset is between 30 and 50 years, with a peak around 40 years for sporadic cases [1,2]. Hereditary forms, associated with genetic syndromes, may present earlier, often between 20 and 30 years [1,2]. Approximately 30-40% are linked to genetic forms, particularly mutations in SDHB, SDHC, SDHD, VHL, RET, and other genes [1,2]. This prevalence underscores the importance of genetic screening, especially in young patients, those with multiple tumors, or those with a family history. In the African context, limited access to advanced imaging and genetic testing may contribute to underdiagnosis, explaining the scarcity of reported cases in local literature. Genetic testing was not performed in our patient. 

Clinically, these tumors typically present with progressive spinal cord compression, without adrenergic symptoms in nonfunctional forms [1,4,5]. Our patient exhibited a similar presentation without headaches, palpitations, or hypertension, confirmed by negative biological tests. Most spinal paragangliomas are non-secreting [1]. The limited data available in the literature confirms the rarity of this location, describing clinical features dominated by spinal cord compression or localized pain, without adrenergic signs [4,5]. Histologically, these tumors are characterized by a nest-like or cord-like architecture of tumor cells (zellballen pattern), surrounded by sustentacular cells and a rich vascular network [1]. Paragangliomas express specific neuroendocrine markers, such as chromogranin A and synaptophysin, used to confirm the diagnosis, while sustentacular cells express S-100 protein, a key marker for their identification. Additional markers, such as tyrosine hydroxylase or dopamine beta-hydroxylase, reflect their chromaffin cell origin [1]. In our case, despite the absence of a typical morphological pattern, immunohistochemistry confirmed the diagnosis. Functional diagnosis relies on measuring catecholamine metabolites, particularly plasma or 24-hour urinary metanephrines and normetanephrines [2]. Certain medications, such as tricyclic antidepressants or antipsychotics, can elevate these levels and lead to false positives [1]. Plasma chromogranin A, though used as a neuroendocrine marker, is less sensitive and specific than catecholamine metabolites [2]. In our patient, no preoperative adrenergic signs were noted, and postoperative biological tests were negative. Radiologically, diagnosis involves conventional and functional imaging. On spinal MRI, paragangliomas typically appear as well-defined, highly vascularized masses with marked contrast enhancement, sometimes associated with hemorrhagic or cystic areas. These features are non-specific, necessitating differentiation from meningiomas, schwannomas, or other hypervascular lesions [1]. Nuclear imaging is valuable for detecting multifocal or metastatic lesions [7]. Several imaging techniques are available, including metaiodobenzylguanidine (MIBG) scintigraphy, 18-FDG PET, 18 F-Dopa PET, and, in some cases, octreotide scintigraphy, although newer tracers like 68Ga-DOTATATE offer increased sensitivity for metastatic lesions [7]. Surgery is the cornerstone of treatment. Complete resection is critical to minimize recurrence risk [5,8,9]. Radiotherapy is indicated postoperatively for incomplete resection or aggressive tumors [8,10]. For benign spinal paragangliomas, stereotactic radiotherapy is also used for unresectable tumors or recurrences [6]. In conventional or intensity-modulated radiotherapy (IMRT), doses typically range from 45 Gy to 50-54 Gy for residual volumes, delivered in 1.8-2 Gy daily fractions [10]. Stereotactic radiotherapy provides symptomatic improvement in 85-87% of patients and rare toxicity (<1% myelopathy) for doses of 14-30 Gy in one to five fractions, offering long-term tumor control (92% at five years, 90% at 10 years) [11]. In our case, the patient received adjuvant radiotherapy of 45 Gy in 1.8 Gy fractions to the D1-D4 region. This case is unusual due to the exceptional intradural extramedullary thoracic location of the paraganglioma in a 27-year-old patient. Postoperative MRI revealed a diffuse and persistent subarachnoid hypersignal, suggestive of tumor residue, with radiologists considering it unlikely that this was solely due to postoperative changes. After multidisciplinary discussion, adjuvant radiotherapy was decided upon to reduce the risk of recurrence. Histological confirmation was not performed for this infiltration.

Lifelong surveillance is recommended. The risk of local or distant recurrence is estimated at approximately 10% at five to 10 years, particularly in patients with genetic forms [1]. This case adds to the limited literature on thoracic spinal paragangliomas in young adults and supports the role of multidisciplinary management.

## Conclusions

Thoracic intradural extramedullary spinal paragangliomas are exceptionally rare, especially in young adults. Accurate diagnosis requires correlation of imaging, histopathology, and immunohistochemistry. Complete surgical resection remains the cornerstone of treatment, while adjuvant radiotherapy may provide additional local control in selected cases. Long-term surveillance is essential to detect recurrence.
